# Contribution of Electronic Medical Records to the Management of Rare Diseases

**DOI:** 10.1155/2015/954283

**Published:** 2015-10-11

**Authors:** Dominique Bremond-Gignac, Elisabeth Lewandowski, Henri Copin

**Affiliations:** ^1^Ophthalmology Department, Amiens University Medical Center, University of Picardie Jules Verne, 80054 Amiens, France; ^2^Ophthalmology Department, University Medical Center Necker Enfants Malades, APHP and CNRS Unit FR3636, Paris V University, 75015 Paris, France; ^3^Department of Medical Information, Amiens University Medical Center, 80054 Amiens, France; ^4^Cytogenetics and Reproduction Biology, Amiens University Medical Center, University of Picardie Jules Verne, 80054 Amiens, France; ^5^EA Hervy, 80000 Amiens, France

## Abstract

*Purpose.* Electronic health record systems provide great opportunity to study most diseases. Objective of this study was to determine whether electronic medical records (EMR) in ophthalmology contribute to management of rare eye diseases, isolated or in syndromes. Study was designed to identify and collect patients' data with ophthalmology-specific EMR. *Methods.* Ophthalmology-specific EMR software (Softalmo software Corilus) was used to acquire ophthalmological ocular consultation data from patients with five rare eye diseases. The rare eye diseases and data were selected and collected regarding expertise of eye center. *Results.* A total of 135,206 outpatient consultations were performed between 2011 and 2014 in our medical center specialized in rare eye diseases. The search software identified 29 congenital aniridia, 6 Axenfeld/Rieger syndrome, 11 BEPS, 3 Nanophthalmos, and 3 Rubinstein-Taybi syndrome. *Discussion.* EMR provides advantages for medical care. The use of ophthalmology-specific EMR is reliable and can contribute to a comprehensive ocular visual phenotype useful for clinical research. *Conclusion.* Routinely EMR acquired with specific software dedicated to ophthalmology provides sufficient detail for rare diseases. These software-collected data appear useful for creating patient cohorts and recording ocular examination, avoiding the time-consuming analysis of paper records and investigation, in a University Hospital linked to a National Reference Rare Center Disease.

## 1. Introduction

Eye health and vision-related conditions related to rare diseases constitute a critical public health issue in countries such as France. The French Government certifies National Reference Centers on rare disease for specific diseases in University Medical Centers in order to improve the quality of health care and support of patients with more than 7,000 listed rare diseases. The concerted efforts by clinicians and scientists interested in these diseases to collect families and refine phenotypic delineation over several decades have led to the discovery of rare genetic diseases. An Italian study of rare diseases estimated the overall raw prevalence at 33.09 per 10,000 inhabitants. Ocular disorders are an example of the most common rare diseases with a prevalence of 4.47 per 10,000 inhabitants [1]. There is an ever-growing need for identification and surveillance of rare eye disease because vision impairment and blindness are major public health problems [2]. Health care systems are increasingly adopting robust electronic health record systems that can not only improve health care but also contain a wide range of data derived from the patient's examination [3]. In ophthalmology, a wide range of data is acquired from patients, requiring the use of specific electronic medical records. However, significant information remains locked in paper text documents, including clinical notes and certain categories of test results. In addition, rare diseases may take a considerable time to accrue in these datasets. A combination of simple free-text searches may be sufficient to obtain informative data from the electronic medical record system for relatively rare cases. A model that is gaining acceptance is clinical innovation based on phenotype information. The information is provided by electronic health records likewise on proposed new therapeutics for patients with rare disease. Typical clinical research is based on purpose-built cohorts or observational studies. Electronic health records are primarily designed to support clinical care, billing, and increasingly other functions such as improvement of quality of life. The present study was designed to determine whether ophthalmology-specific electronic medical records are useful for the management of rare eye diseases or rare syndromes with eye involvement.

## 2. Methods

This study was designed to identify and collect data from patients with specific rare eye diseases, registered with ophthalmology-specific electronic medical records at an eye center. Five rare diseases were selected on the basis of their rarity and the center's particular expertise. The five diseases selected for this study were congenital aniridia, Axenfeld/Rieger syndrome, Blepharophimosis-Epicanthus-Ptosis Syndrome (BEPS), Nanophthalmos, and Rubinstein-Taybi syndrome.

### 2.1. Patient Data

The study was carried out in the Department of Ophthalmology at University Medical Center of Amiens. The names and data of patients with the five selected rare eye diseases were collected. The patients were seen at the Rare Eye Diseases Reference Center in Amiens linked to the National Reference Rare Eye Disease Center in Paris from 1 January 2011 to 31 December 2014. All patients attending an outpatient visit and patients undergoing inpatient or outpatient surgery were included.

### 2.2. Electronic Medical Records

An ophthalmology-specific electronic medical record software was installed in the DX Care institutional electronic health records at University Medical Center of Amiens in January 2010. A descriptive, longitudinal record is created for each patient to describe the events at each meeting with the patient. The record describes all visits attended by the patient, case histories, diagnoses, medications, medical and surgical procedures, tests, and investigation results. The main types of information available from EMRs are laboratory results and vital signs, provider documentation, documentation from clinical ocular examinations and tests, medication records, and tests' results such as visual field, corneal OCT, corneal topography, pachymetry, tear osmolarity, macular OCT, and electrophysiology. Each user is assigned a personal user name and password. The system ensures a high level of security and is accessible to clinicians working at the center including residents and fellows who enter patient data as well as the center's health care providers.

### 2.3. Methods

**Figure 1:**
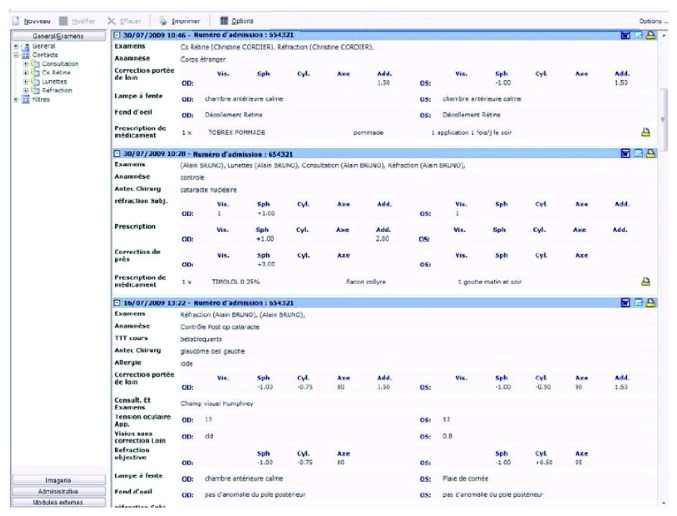
Softalmo software, ophthalmological medical record main page.

During the period from 1 January 2011 to 31 December 2014, patients were examined at the University Medical Center of Amiens and their data were recorded in the specific electronic medical record (software for ophthalmologists Softalmo Corilus, France). Physicians, nurses, and medical secretaries log onto the software with an individual login and password or an external professional card reader. The software comprises the patient's administrative data and the patient's detailed ocular examination. The EMR ocular examination includes the following fields: purpose of the consultation, history of symptoms and clinical signs, treatment, allergy, patient's medical history, family medical history, ocular motility, lids and lacrimal drainage, visual acuity (noncorrected, corrected, with cycloplegia, and optical correction), refraction, slit lamp examination, intraocular pressure, fundus examination, diagnosis, conclusion, orthoptic examination, and remarks (Figure 1). The electronic medical record is also linked to all of the new ocular investigations, such as IOL Master A scan, pachymetry, anterior segment OCT, posterior segment OCT, all types of corneal topography, all types of visual field technologies, electrophysiology, fundus photography, and angiography. Five specific rare eye diseases were studied in the patients' files. “Ophtalmo Query” from Softalmo (Corilus) computer search software was used to identify all terms for each rare disease in all text fields of the ocular electronic medical record.

## 3. Results

From 1 January 2011 to 31 December 2014, 135,206 patients attending a consultation at University Medical Center of Amiens and 16,039 patients undergoing inpatient or outpatient surgery were included using ophthalmology-specific electronic medical records Softalmo (Corilus). The five rare eye diseases were identified from the files of outpatients of the center by using the search function of the software (Ophtalmo Query). The results are reported in Table 1. The frequency of visits for each rare disease is reported in Table 1. For each rare disease selected with patients' names corresponding patient's data are collected. All visual investigations performed were available at the same time with the ophthalmology-specific EMR. The search software identified 29 patients with congenital aniridia, 6 patients with Axenfeld/Rieger syndrome, 11 patients with Blepharophimosis-Epicanthus-Ptosis Syndrome (BEPS), 3 patients with Nanophthalmos, and 3 patients with Rubinstein-Taybi syndrome. The frequency of the visits was different for each rare disease. In 4 years, aniridic patients had a mean of 2.1 visits, Axenfeld/Rieger syndrome patients had a mean of 8.4 visits, BEPS patients had a mean of 3 visits, Nanophthalmos patients had a mean of 2.3 visits, and Rubinstein-Taybi syndrome patients had a mean of 3.3 visits. The EMRs for each of these patients were reviewed to confirm the accuracy of the diagnosis. A combination of simple free-text searches followed by manual chart review may be sufficient for relatively rare case reports.

## 4. Discussion

Electronic medical records provide a number of advantages in terms of medical care, including decreased paperwork, improved administrative efficiency, reduction of time-consuming medical record data collection, and decreased data processing burden [4]. The use of nonspecialized electronic medical records can present serious clinical drawbacks for eye care providers, as the used system may not be adequate documentation of ophthalmological examinations and care. Typically, patient questionnaires and/or analysis of research staff are used to determine the patient's phenotypic traits. These very costly and time-consuming models are common and represent only a specific point in time. Our study demonstrated that the ophthalmology-specific EMR system used in the Ophthalmology Department of University Medical Center provides accessible and reliable information. The ophthalmic EMR is specific and can contain ocular information not easy to include in a general EMR. This study emphasizes the value of EMR for data collection in rare eye diseases. The use of studies of EMR for data collection in rare eye diseases will be done with approval by the Human Research Ethics Committees. Specific and adapted ophthalmological electronic medical record software can provide real-time data including all ocular tests and ophthalmological investigations [5]. This automated data extraction is much less time-consuming than traditional chart-based reviews of medical records [6]. In addition, electronic medical records data entered by care providers during the medical care process are objective, whereas survey data based on patients' self-reports are not objective. A key advantage of ophthalmology-specific EMRs is that they allow collection of phenotype information as a by-product of routine health care. Moreover, this data collection becomes more extensive with time and is continually refined as new information confirms or excludes a diagnosis in a given individual. Data concerning disease, response to treatment, and laboratory test data are collected during the patient's lifetime. Aggregation of this information can allow larger sample sizes for certain rare diseases and is particularly useful for patients with a rare disease [7]. EMR requires a significant institutional investment and ongoing financial, ethical, and logistical support to operate effectively. Even the implementation of an electronic medical record system that meets these particular needs may lack the charting function required to document eye examinations and care. Implementation of an EMR system is also likely to involve an initial loss of income due to the time devoted by health care providers to installing, learning, and personalizing the system. Funding is also required for staff training and acquiring resources to use and maintain the system [1]. However, eye care professionals need to be involved in the EMR design process in order to ensure effective workflow integration and the inclusion of EMR components specific to eye care. Finally, integration of the system into daily care appears much easier under these conditions. EMR is not yet universally available but provides major advantages for equipped centers for the management of rare diseases. The frequency of the visits of patients with rare eye disease or syndrome with eye involvement must be analysed for improved health management or customized patient pathways for rare eye diseases. EMRs can help improve eye health in rare diseases by analyzing data derived from patient cohorts. EMRs could possibly facilitate information sharing locally and in the department and with national university medical centers and national rare disease centers once this type of data exchange is supported.

## 5. Conclusion

Finding information on rare diseases is a data mining problem due to the small number of patients and the complexity of the disease. Processing a large number of records requires an automated extraction and classification process. In conclusion, our study demonstrated the reliable information on targeted rare diseases provided by the ophthalmology-specific EMR system. A cohort of patients can be constructed for each rare disease, which can therefore be useful for rare disease public health centers. The collection of accurate patient data constitutes a major challenge. The value of data for a given rare disease of interest is therefore essential to improve clinical research. The use of specific EMR data may allow improved surveillance of rare diseases, including surveillance of eye health and vision-related conditions. The sharing of data with all centers caring for patients with a rare disease will ultimately support clinical research and innovation.

## Figures and Tables

**Table 1:** Rare disease EMR research.

Rare diseases	Patients of active file	Number of visits (2011–2014)
Aniridia	29	61
Axenfeld-Rieger syndrome	5	42
BEPS	11	33
Nanophthalmos	3	7
Rubinstein-Taybi syndrome	3	10
